# Comparing population trend estimates of migratory birds from breeding censuses and capture data at a spring migration bottleneck

**DOI:** 10.1002/ece3.7110

**Published:** 2020-12-19

**Authors:** Ivan Maggini, Massimiliano Cardinale, Andrea Favaretto, Petr Voříšek, Fernando Spina, Francesco Maoret, Andrea Ferri, Sara Riello, Leonida Fusani

**Affiliations:** ^1^ Austrian Ornithological Centre Konrad‐Lorenz Institute of Ethology University of Veterinary Medicine Vienna Wien Austria; ^2^ Department of Aquatic Resources Institute of Marine Research Swedish University of Agricultural Sciences Lysekil Sweden; ^3^ Cisca ‐ Italian Centre of Research and Conservation of the Environment Cassino Italy; ^4^ Czech Society for Ornithology Prague Czech Republic; ^5^ Department of Zoology and Laboratory of Ornithology Faculty of Science Palacky University Olomouc Olomouc Czech Republic; ^6^ Institute for Environmental Protection and Research (ISPRA) Ozzano dell'Emilia Italy; ^7^ Riserva Naturale Statale “Isole di Ventotene e S. Stefano” Ventotene Italy; ^8^ Department of Behavioural and Cognitive Biology University of Vienna Wien Austria

**Keywords:** bird ringing, migratory bottleneck, PECBMS, Ponza, population indices

## Abstract

Europe has a well‐established network of breeding bird monitoring that is used to produce supranational indices of population trends for many species. However, a comparison of breeding bird censuses with other methods may be beneficial to confirm the validity of such indices. The aim of this study was to assess the value of standardized capture data of migratory birds at migration bottlenecks as an indicator of the effective breeding populations. One limitation to this method is that several populations are co‐occurring at these bottlenecks and their catchment areas need to be clearly identified to allow extrapolation of population indices. Here, we used standardized trends in capture numbers of 30 species on the island of Ponza, a migration bottleneck in the central Mediterranean, and compared them to population trends estimated in the putative catchment breeding areas between 2005 and 2016. The catchment areas were identified through the analysis of ring recoveries during the breeding season of birds passing through Ponza. Our results show an agreement between the population trends observed on Ponza and those in the breeding areas in 15 out of 30 species. The correlations were strongest in species with a more robust definition of the catchment areas, that is, species with more than 10 recoveries, and for which the recoveries were most likely of breeding birds. The main reason for disagreement between the two indices in the remaining species might be related to different intensity of sampling in different areas. This issue can be solved by further developing monitoring projects in underrepresented countries, as well as by intensifying monitoring through ringing, both in the breeding grounds and at migration bottlenecks. These results show that spring migration monitoring at bottlenecks has the potential to provide a valuable complement and an independent control of breeding bird surveys, allowing raising early warnings of population declines and contributing to their conservation.

## Graphical abstract

We compared trends of 30 migrant Passerine bird species on passage through a migration hotspot with population trends in their breeding areas. We found good agreement between trends in half of the species. This shows how integrating different count methods could benefit monitoring and conservation.

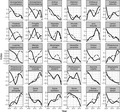

## 1 INTRODUCTION

Monitoring changes in bird populations is fundamental to understand ecological processes such as changes in the environment (Järvinen & Väisänen, 1979; Morrison, 1986) and for conservation (Baillie, 1991; DeSante & Rosenberg, 1998). In Europe, most countries contribute census data to a concerted program, the PanEuropean Common Bird Monitoring Scheme (PECBMS, https://pecbms.info/). The PECBMS collates national indices obtained from breeding bird censuses to produce supranational and continental trend indices for a large number of common species (Gregory et al., 2008; van Strien et al., 2001; Voříšek et al., 2008). Breeding bird censuses use different counting techniques (Bibby et al., 2000; Ralph et al., 1993), and the choice of count areas might not be entirely random, as a consequence of, for example, scarce accessibility to some sites. In addition, the precision of species indices might be affected by problems related to unknown detectability. Although there are methods to account for different species detectability (Buckland et al., 2001), most survey schemes do not adjust for it on a routinely basis (Gregory & van Strien, 2010; Johnson, 2008). Despite these limitations, these indices provide important data to assess population trends and to confirm the role of birds as indicators of environmental change (Gregory et al., 2019). However, to date there has been no attempt to compare population indices from generic monitoring schemes with indices obtained by other, independent, methods to assess their validity at an international level.

Mist netting is an established method to assess population size and demographic parameters of many species (Dunn & Ralph, 2004). One advantage of mist netting over other methods is that it allows to sample species that might be difficult to detect in the field, because of their inconspicuousness or because their breeding habitats are hard to reach and/or sample (Rappole et al., 1998; Wang & Finch, 2002). Mist netting also offers the chance to gather a wealth of side data about the individuals, such as sex, age, breeding condition, physiological condition, genetics, etc. On the other side, its efficiency varies among species and sometimes age and sex classes (Nur et al., 2004; Wang & Finch, 2002), it can be heavily dependent on weather (Simons et al., 2004), and especially in long‐term studies, it requires a great amount of standardization over time to provide reliable estimates (Baillie & Schaub, 2009; Hussell & Ralph, 2005; Kaiser & Berthold, 2004; Porzig et al., 2011).

Breeding populations can be sampled in the areas where reproduction occurs, but good estimates can also be obtained from ringing stations active during the migratory season (Berthold, 2004; Crewe et al., 2015; Dunn et al., 1997; Karlsson et al., 2005; Lloyd‐Evans & Atwood, 2004; Osenkowski et al., 2012; Rimmer et al., 2004). While autumn migration totals can reflect productivity (in terms of young individuals produced during the previous breeding season), spring migration totals provide an estimate of the population going back to the breeding sites, therefore reflecting the effective population size (Dunn, Hussell & Adams, 2004; Dunn, Hussell, Francis et al., 2004). The main problem with inferring population size from migration ringing totals is the intermix of different populations at stopover sites, where most of the ringing usually occurs. Ringing stations and bird observatories are usually located at migration bottlenecks that attract large numbers of individuals from different populations and species (Cardenas‐Ortiz et al., 2020; Dunn, 2016). The catchment areas of birds present at a given stopover site at a given time can only be inferred from indirect methods, such as ringing recoveries (Fiedler et al., 2004; Thorup et al., 2014), stable isotope analysis of feathers (Hobson et al., 2015), or genetic analysis (Clegg et al., 2003).

Previous attempts of comparing long‐term trends in breeding population size with migration trapping totals have obtained mixed results: many studies found good agreement in most species while other studies did not, at least for some species at some sites (Dunn, Hussell, Francis et al., 2004). Most of these analyses were conducted in North America, where standardized mist netting has been conducted at several study sites with similar methods since the 1990s (Hussell & Ralph, 2005). The distribution of many species in the boreal forest makes it particularly important to sample population trends at locations different from the breeding areas, since these are impossible or hard to reach. The North American estimates obtained from trapping totals and migration counts (Hussell & Ralph, 2005) provide useful measures of breeding populations for most species (Crewe et al., 2008). Similar highly standardized projects are being conducted in Europe (Bairlein, 1995; Berthold et al., 1998; Pedrini et al., 2008; Spina, 2011; Spina et al., 1993), but these data have rarely been used to track fluctuations of the breeding populations (but see Berthold, 2004).

Within Europe, an important bottleneck during spring migration for species wintering in Africa is certainly represented by the small islands in the Mediterranean Sea (Gargallo et al., 2011; Spina et al., 1993). A standardized monitoring of spring migration started in the late 1980s and included a large number of stations, some of which have been active for more than 30 years. The high number of species and individuals trapped on these islands makes them ideal candidates to monitor population trends of several bird species in Europe. In this study, we focused on data of the 30 most commonly captured species on one small Italian island (Ponza, in the Tyrrhenian Sea), where standardized mist netting has been conducted continuously for almost 20 years. The aim of the study was to assess the fit between the population trend estimates of these species on Ponza with those calculated using the PECBMS supranational indices. To identify the catchment areas of different species passing on Ponza, we analyzed ring recoveries of birds found during the breeding season. We then adapted the PECBMS indices to match the calculated area, and compared them to the trends observed on Ponza. A good fit would confirm the usefulness of monitoring at this migration bottleneck as an estimate of population changes in the breeding areas, thus providing an additional, complementary tool to identify early signs of change in breeding populations and promote conservation measures in the specific catchment areas.

## 2 MATERIALS AND METHODS

### 2.1 Study site and ringing operations

This study was conducted on Ponza, a small island in the Tyrrhenian Sea (9.87 km^2^) located about 50 km off the Western coast of Italy (40°55′N, 12°58′E), where we have been monitoring spring migration through capture and ringing since 2002 (www.inanellamentoponza.it). Ponza is located along one of the main Mediterranean migratory routes and attracts large numbers of African‐European migratory landbirds during spring migration (Maggini, Trez et al., 2020). The bird capture season started in March in most of the years and ended in May (see Table S1 for start and end dates for every year). Birds were captured with an average of 227 m of mist nets that were opened every day except for days with heavy rain or strong winds (>15 knots). These conditions occurred on <1% of the days during the study period. The mist nets were checked hourly from dawn until one hour after dusk. We kept the net brand (Lavorazione Reti Bonardi, http://www.vbonardi.it/) and model (2.4 m height, 16 mm mesh size) constant throughout the entire study period. Vegetation height was kept constant throughout the entire study period. After being captured, birds were ringed and measured using standard procedures (Bairlein, 1995). For this analysis, we selected the 30 most abundant species in the period between 2005 and 2016 (Table 1), during which the ringing procedures were fully standardized.

**TABLE 1:** Summary of the 30 most abundant species on Ponza used for the analysis of population trends in this study

Species	Common name	Total individuals captured	Average captures per year (min/max)	Median passage date
*Acrocephalus arundinaceus*	Great Reed Warbler	410	23 [4, 58]	124.2
*Acrocephalus schoenobaenus*	Sedge Warbler	1,162	65 [17, 236]	127.1
*Anthus trivialis*	Tree Pipit	2,193	122 [19, 202]	108.4
*Delichon urbicum*	House Martin	1,295	72 [9,136]	114.5
*Erithacus rubecula*	European Robin	13,044	725 [1, 2,599]	86.5
*Ficedula albicollis*	Collared Fylcatcher	1,548	86 [1, 279]	112.7
*Ficedula hypoleuca*	Pied Flycatcher	10,312	573 [65, 914]	115.8
*Fringilla coelebs*	Common Chaffinch	357	22 [0,58]	77.6
*Hippolais icterina*	Icterine Warbler	6,608	367 [45, 583]	109.4
*Hirundo rustica*	Barn Swallow	507	28 [1, 67]	105.3
*Jynx torquilla*	Eurasian Wryneck	676	38 [17, 72]	121.0
*Lanius senator*	Woodchat Shrike	1,811	101 [11, 214]	105.3
*Luscinia megarhynchos*	Common Nightingale	736	41 [13, 114]	120.7
*Merops apiaster*	European Bee‐eater	8,654	481 [46, 1,052]	130.4
*Muscicapa striata*	Spotted Flycatcher	18,871	1,048 [216, 2,922]	134.3
*Oenanthe oenanthe*	Northern Wheatear	2,097	117 [21, 212]	105.7
*Oriolus oriolus*	Golden Oriole	1,404	78 [19, 182]	123.4
*Phoenicurus ochruros*	Black Redstart	1,972	110 [0, 397]	83.1
*Phoenicurus phoenicurus*	Common Redstart	5,474	304 [45, 726]	110.4
*Phylloscopus collybita*	Common Chiffchaff	3,809	212 [1, 589]	86.9
*Phylloscopus sibilatrix*	Wood Warbler	14,784	821 [159, 1,430]	116.4
*Phylloscopus trochilus*	Willow Warbler	12,673	704 [83, 1,321]	110.0
*Saxicola rubetra*	Whinchat	16,980	943 [455, 1,647]	119.5
*Streptopelia turtur*	European Turtle Dove	564	31 [16, 49]	123.0
*Sylvia atricapilla*	Blackcap	2,303	128 [4, 574]	98.4
*Sylvia borin*	Garden Warbler	49,713	2,762 [581, 5,967]	130.2
*Sylvia cantillans*	Subalpine Warbler	8,019	446 [29, 1,089]	97.2
*Sylvia communis*	Greater Whitethroat	30,496	1,694 [500, 3,594]	121.3
*Turdus philomelos*	Song Thrush	1,828	102 [0, 477]	82.8
*Upupa epops*	Eurasian Hoopoe	458	25 [1, 64]	91.9

Note

Total captures between 2005 and 2016, average captures per year and median passage dates (Julian day: January 1st = day 1) on Ponza are given.

### 2.2 Estimate of yearly numbers of passing migrants

For every species and year, we calculated a standardized Catch Per Unit Effort (CPUE; expressed as the number of birds caught per hour and meter of net deployed) index (hereafter “Ponza index”), which reflects the year trend proportional to the bird density passing through Ponza. To account for the unbalanced sampling design between years and days (expressed as Julian day) and remove these effects from the nominal CPUE, we used generalized additive models (GAMs, Hastie & Tibshirani, 1990, and see Maunder & Punt, 2004 for a useful review on different standardization approaches). The classic CPUE lognormal model (where the natural logarithm of CPUE is set as the response variable) cannot be mathematically applied in the case of including zero‐catch data. Thus, we utilized the Tweedie distribution model (Tweedie, 1984; Wood et al., 2016). Unlike lognormal or gamma distributions, the Tweedie distribution admits zero values and does not require transformation of the data. The following is the GAM model formulation:

$$\text{CPUE}=\beta +\left(\text{year},\;\text{day}\right)+\varepsilon$$

where *β* is a constant and *ε* is an error term. Both year and day were treated as a factor in the analysis. The interaction term between year and day was included in the model to account for significant changes in the time of passage of the different species during the period analyzed (Maggini, Cardinale et al., 2020).

### 2.3 Geographical analysis of recapture data

To determine the origin of the populations of birds passing through Ponza, we used a dataset of ringing recoveries of individuals of the 30 species from which we calculated the Ponza index caught between 1989 and 2016 in the Pontine Islands (Ventotene, Ponza, and Zannone, which are islands in the same archipelago where bird ringing occurred in the last 30 years) and included in the EPE National Database of Italian ISPRA (www.epe.isprambiente.it). We extended the database to a longer period of time and to the neighboring islands to ensure a larger sample of recovery data, assuming that the populations of origin would not differ within this small range. We created a database of the Pontine Islands recoveries including all birds ringed on the islands and found elsewhere and foreign birds found on the islands as an ESRI *shapefile* (Mitchell, 2005). For each individual, we converted latitude and longitude of trapping and recovery site, expressed in geographical decimals degrees, into UTM 32 Datum WGS 84 format in order to measure distances and surfaces on a metric base.

To ensure as much as possible that recovery data were originating only from birds that were actually in their breeding area, and not on migration, we considered only recoveries of individuals that were caught between mid‐March and mid‐September. This approach was a compromise between restricting the period too much and therefore reducing the number of recoveries available for successive analysis, and considering a wider period, running the risk of including individuals that were still migrating and thus not being actual breeders. Indeed, in Europe, the breeding period for many species mainly coincides with the months of June and July, but can vary considerably depending on several factors, for example, latitude (e.g., Sanz, 1997, 1998). However, when considering only recoveries that occurred in this narrow time period (June and July), the dataset returned only 42 recoveries, which was an insufficient sample for this analysis. Therefore, we expanded the subset by including recoveries between mid‐March and mid‐September while manually discarding individuals that appeared to be most likely migrating, considering the time and location of the recovery (listed in Table S2). With this approach, our dataset contained 141 valid recoveries (Table 2). For two species, Willow Warbler (*Phylloscopus trochilus*) and Barn Swallow (*Hirundo rustica*), with sufficient recoveries in the period June‐July (*N* = 8 and *N* = 6, respectively), we compared the result obtained with this subset of data with the one obtained with a larger sample (shown in Table S2).

**TABLE 2:** Breeding areas of birds recaptured from or on the Pontine Islands between 1989 and 2016 and used for the calculation of the catchment areas of birds migrating through the area during spring

Species	Central & East Europe	North Europe	South Europe	South East Europe	West Balkan	West Europe	Total
*Acrocephalus arundinaceus*	4						4
*Acrocephalus schoenobaenus*	1	2				1	4
*Acrocephalus scirpaceus*			1		3	1	5
*Erithacus rubecula*	2	1					3
*Ficedula albicollis*	2	1				1	4
*Ficedula hypoleuca*	1	1			1	2	5
*Hippolais icterina*	3	1		1		1	6
*Hirundo rustica*	3		3		6	7	19
*Merops apiaster*	1				1		2
*Oenanthe oenanthe*					1		1
*Oriolus oriolus*				1			1
*Phoenicurus ochruros*	1						1
*Phoenicurus phoenicurus*	3					2	5
*Phylloscopus collybita*	3	5				3	11
*Phylloscopus sibilatrix*		1					1
*Phylloscopus trochilus*	7	17				6	30
*Saxicola rubetra*	1						1
*Sylvia atricapilla*	3	1			1	6	11
*Sylvia borin*	8	6			1	1	16
*Sylvia communis*	7	3				3	13
*Upupa epops*	2						2

Each individual bird was assigned to one of six geographic regions as defined by the PECBMS. However, some of the individual birds were ringed or recovered in countries not assigned to any of the regions as not all European countries where ringing activities are carried out are included in the PECBMS indices. Those individuals were manually assigned to one of the PECBMS regions as follows:

- Birds trapped or recaptured in Croatia, Serbia, Montenegro, Bosnia Herzegovina, and Northern Macedonia were assigned to PECBMS region “West Balkan”;
- Birds trapped or recaptured in the western part of Russia (i.e., Kaliningrad) were assigned to the PECBMS region “Central‐East Europe.”

We analyzed the geographical distribution of the recoveries at the species level using Directional Distribution Analysis (DDA; Wang et al., 2015). DDA estimates an ellipse of the spatial distribution of the observations through the standard deviation of the distance between the observations in order to estimate the central tendency of the observations (i.e., centroid of the spatial distribution of the recoveries), the dispersion, and the directional trend if present. We conducted DDA at the species level for all species with three or more recoveries, the minimum sample size to construct an ellipse (Wang et al., 2015). For each species, we measured the centroid of the spatial distribution of the recoveries as well as the statistical ellipses that encompass the putative area of the distribution of the recoveries. The estimated ellipses were then overlapped with the PECBMS regions to calculate the area of the ellipses (expressed in proportion of the estimated total area of the ellipses) covering each of the PECBMS regions. These proportions were then used to weigh the regionally specific PECBMS indices by species (i.e., a species‐specific ellipse) and calculate a single area‐weighted PECBMS index for the period 2005–2016 to be compared with the Ponza index. For example, if the area of the ellipse was 80% within the North Europe region and 20% over the Central Europe region, the weighted PECBMS index was obtained from adding the North Europe index multiplied by 0.8 to the Central Europe index multiplied by 0.2. For species with less than three recoveries, a general putative ellipse of the distribution was estimated using the recoveries of all species lumped together.

### 2.4 Correlation analysis between Ponza index and PECBMS area‐weighted population index

Species population indices in the PECBMS regions were obtained from the calculation of European species trends and indices for the period 2005–2016 (https://pecbms.info/methods/pecbms‐methods/). For species for which the index of one or more PECBMS regions was not available, the weight of the missing region was redistributed between the other regions maintaining the same proportion between the regions with data. Using the weight matrix, an area‐weighted PECBMS index was estimated for every species considered in the analysis. The area‐weighted PECBMS index was then centered subtracting the mean and dividing it by its standard deviation and finally smoothed to extract the long‐term trend. For the Ponza index, we performed LOESS smoothing with the geom_smooth() function from the ggplot package in R to extract the long‐term trend and make the two indices comparable. For each species, we analyzed the slopes of the linear correlation between the area‐weighted PECBMS index and the Ponza index in the years between 2005 and 2016, to test the hypothesis that the Ponza index estimated during spring migration is a measure of the size of the bird population estimated by PECBMS in the same year.

## 3 RESULTS

### 3.1 Geographical distribution of recaptures during the nesting period

**FIGURE 1:**
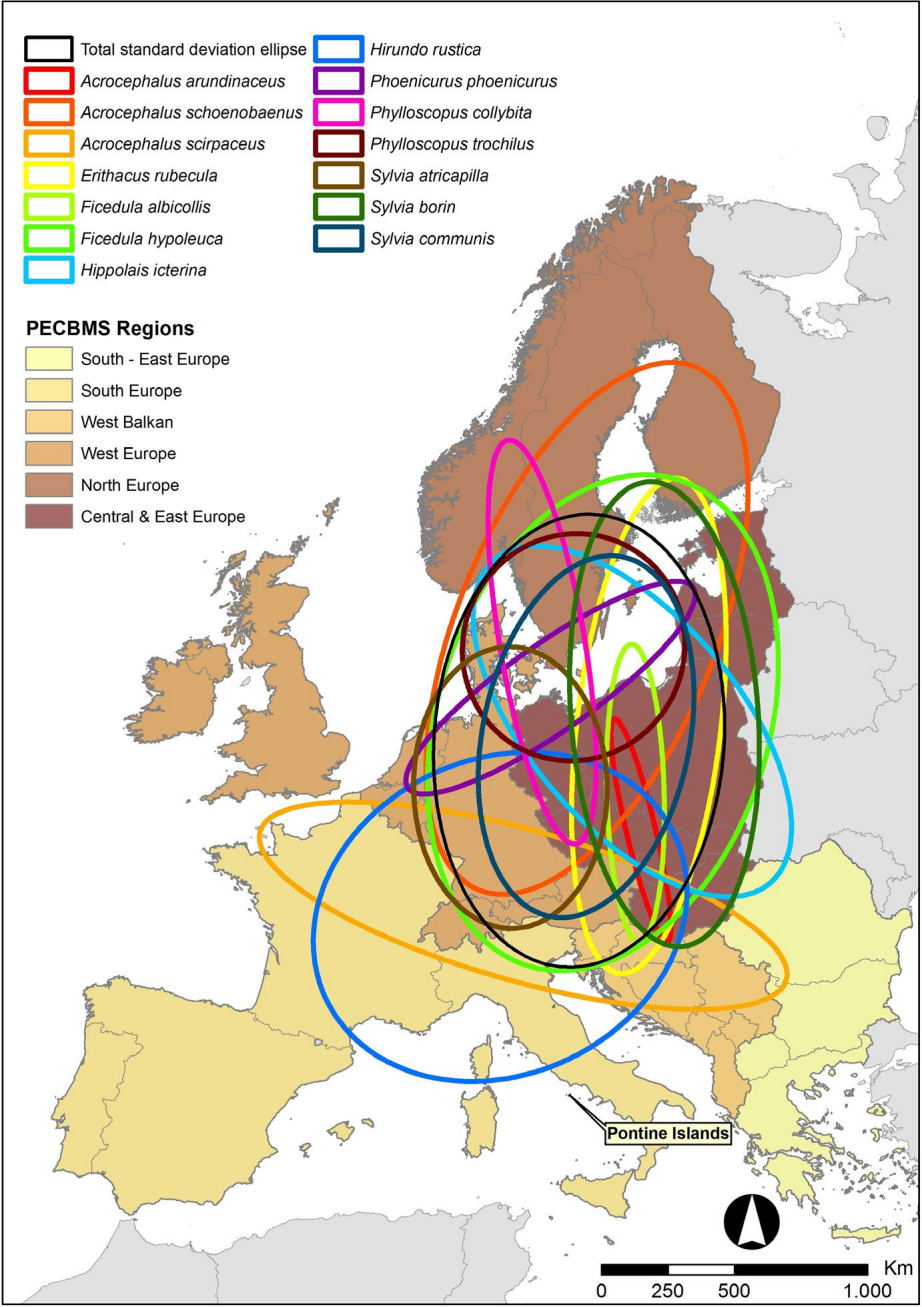
Breeding origins estimated using standard deviational ellipses of recoveries made during the breeding period for species with three or more recoveries

The main breeding areas for Ponza birds were “Central & East Europe” (37% of recoveries), followed by “North Europe” (28%) and “West Europe” (24%) (Table 2). Willow warblers were the species with most recoveries (30) followed by Barn Swallows (19), Garden Warblers (*Sylvia borin*) (16) and Whitethroats (*Sylvia communis*) (13) (Table 2). The spatial distribution of all recoveries is shown in the Appendix (Figure S1). For 14 species, there were three or more recoveries and we could calculate a species‐specific standard deviational ellipse (Figure 1). The centroids for these species are summarized in the Appendix (Table S3).

### 3.2 Correlation analysis between area‐weighted PECBMS index and the Ponza index

**FIGURE 2:**
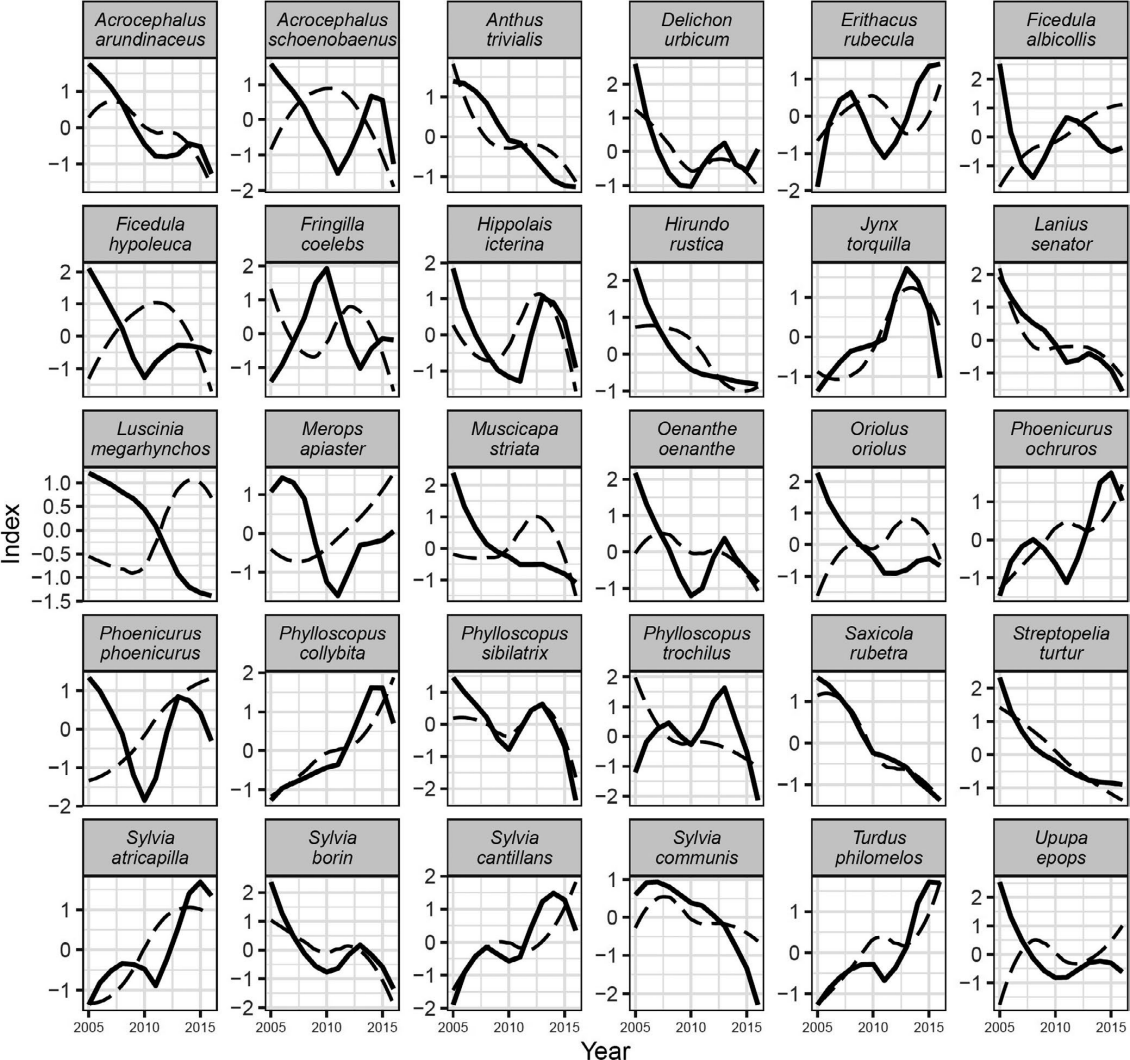
Ponza index (bold line) and area‐weighted PECBMS index (dashed line) plotted for 30 species over the study period between 2005 and 2016

Area‐weighted PECBMS index and Ponza index for 30 species are shown in Figure 2. The results of the correlation analysis between the two indices are shown in Table 3. We estimated a significant correlation with a positive slope for 15 species. For one species (Black Redstart, *Phoenicurus ochruros*), the correlation was nearly significant with a positive slope. In three species (Common Nightingale *Luscinia megarhynchos*, Golden Oriole *Oriolus oriolus*, and Hoopoe *Upupa epops*), there was a significant correlation with a negative slope, that is, an increase of birds passing through Ponza Island corresponded to a decrease in their breeding areas in Europe.

**TABLE 3:** Correlations (with slope and significance level) between Ponza index and area‐weighted PECBMS index

Species	Slope	*p*
*Acrocephalus arundinaceus*	1.01	.006
*Acrocephalus schoenobaenus*	−0.29	.413
*Anthus trivialis*	1.06	<.001
*Delichon urbicum*	0.94	.014
*Erithacus rubecula*	0.50	.399
*Ficedula albicollis*	−0.48	.161
*Ficedula hypoleuca*	−0.60	.059
*Fringilla coelebs*	−0.61	.093
*Hippolais icterina*	0.60	.105
*Hirundo rustica*	0.97	.006
^a^	0.73	.045
*Jynx torquilla*	0.93	<.001
*Lanius senator*	1.01	<.001
*Luscinia megarhynchos*	−1.14	<.001
*Merops apiaster*	−0.41	.324
*Muscicapa striata*	−0.14	.758
*Oenanthe oenanthe*	0.75	.223
*Oriolus oriolus*	−1.02	.003
*Phoenicurus ochruros*	0.68	.070
*Phoenicurus phoenicurus*	−0.11	.731
*Phylloscopus collybita*	0.92	<.001
*Phylloscopus sibilatrix*	1.32	<.001
*Phylloscopus trochilus*	0.15	.697
^a^	−0.45	.210
*Saxicola rubetra*	1.04	<.001
*Streptopelia turtur*	0.96	<.001
*Sylvia atricapilla*	0.75	.006
*Sylvia borin*	0.96	.003
*Sylvia cantillans*	0.82	.012
*Sylvia communis*	1.77	.005
*Turdus philomelos*	0.96	.001
*Upupa epops*	−0.99	.007

^a^ For *H. rustica* and *P. trochilus* indicate results using only the subset of recoveries from June to July.

When considering only recoveries from June‐July in Willow Warblers and Barn Swallows, our result did not change substantially. The correlation between the two indices remained significantly positive in the Barn Swallow, while there was no significant correlation in the Willow Warbler (Table 3). For both species, however, the correlation was less strong than when using the extended dataset.

## 4 DISCUSSION

Estimating population trends of migratory birds in large areas is challenging and requires a huge effort in terms of observers and time. Several European countries have well‐established protocols for monitoring their breeding bird populations, while others may lack sufficient manpower or funding. This is especially true for countries in the East of Europe (see information about national programs and number of collaborators on the PECBMS website: https://pecbms.info/country/). Monitoring and conservation of migratory birds in these countries might benefit from trend estimates based on trapping at spring migration bottlenecks, like the ones from small islands in the Mediterranean Sea. Our study identified the catchment areas of birds captured on the island of Ponza to be centered mostly in Central‐Eastern Europe. There was good agreement between population trends in Ponza and those reported from the breeding areas in half of the species. For these species, capture numbers on Ponza can be an ideal complement to breeding bird censuses.

Our study shows that ringing totals at a migratory bottleneck can be a powerful conservation tool for monitoring a large number of species. Ringing data can be used as an independent control of trends based on monitoring data, while providing additional data such as age structure or individual condition of birds. Moreover, monitoring at migration bottlenecks is very efficient in identifying changes in the phenology of migrants (Maggini, Cardinale et al., 2020). As bird surveys in the breeding grounds have standardized protocols allowing fieldworkers to do their count in specific time windows during the breeding season, a change in phenology could produce a bias because of a mismatch between the timing of arrival and peak activity of the birds and the counting period (Massimino et al., 2020). The use of migration data can help inform these procedures and correct the bias.

It is important to note that indices as those estimated in Ponza are usually available immediately after the migratory season, thus providing an anticipation of the same year's breeding population, while generally data from different types of breeding bird censuses are published after a couple of years. This is true for supranational indices produced within PECBMS as those used here, although at national level, most of the national schemes have their annual updates ready generally at the end of the breeding season. However, it is also important to note that, given the nature of population indices, year‐to‐year changes are affected by various short‐term factors and long‐term trend data are needed for conservation purposes. While readily available yearly indices as those estimated here can be valuable to flag drastic and sudden declines in a breeding population (i.e., very early warning signals), the value of these indices increases considerably when calculated over a long time range.

While the good association between indices might support the use of either one of the methods for assessing population changes, it is important to note that trends in Ponza did not always match the trends in the breeding areas. However, we found a good match in five of the six species with more than 10 recoveries in the last 30 years. The only exception was the Willow Warbler, for which we did not find a matching trend despite it being the species with most recoveries. One possibility is that some of the recoveries might have been of birds that were still migrating. The Willow Warbler is the species with the northernmost centroid, and Northern populations start breeding later than May 15th, and possibly are on migration again in August (Hedlund et al., 2015). Therefore, our ellipse might have been shifted to an area that did not represent exactly the breeding catchment area for this species, possibly over representing the Central & East European populations. In fact, the ellipse shifted slightly toward the North when considering only recoveries in the months of June and July. Nevertheless, recalculating the correlation did not yield a better match of the indices. This might be due substantially lower sample size. In species with less than 10 recoveries, there was a mix of matching and non‐matching trends which could not be explained by differences in the ecology or distribution of the species. For some of these species, this mismatch is probably caused by an inaccurate estimate of the putative catchment area. Taken together, these results point out the importance of a precise assessment of the catchment area of the migratory populations passing through a bottleneck (Osenkowski et al., 2012), and of a careful selection of recoveries that can be attributed with certainty to breeding birds. In our sample, we decided to include a few species that did not fit these criteria since this allowed us to underline the importance of these aspects. Our study does not allow determining whether a mismatch between indices is revealing inaccuracy of either of the methods used. While a low number of recoveries or an inaccurate estimate of the catchment area could influence the estimates based on ringing data, this method is likely to provide a more homogenous sampling of the breeding populations across their range. Breeding bird censuses might not cover such areas as homogenously, thus resulting in differences in sampling accuracy between the two methods. Future research should address these issues to allow determining which method is the most accurate. We considered the possibility that a shift in phenology in the breeding grounds would affect population estimates (Massimino et al., 2020). If this was true, we would expect a larger mismatch in species that are advancing their arrival in Europe to a larger extent. We cannot find support for this interpretation, since out of the five species that are showing a significant advance in arrival on Ponza (Maggini, Cardinale et al., 2020), only two species (European Robin *Erithacus rubecula* and Common Redstart *Phoenicurus phoenicurus*) show a mismatch in their population indices between Ponza and the breeding grounds. However, the effects of shifted phenology on breeding bird estimates might become more apparent over a longer study period.

To date, we still rely on large numbers of recaptures from bird ringing, since new technologies such as geolocators or GPS‐transmitters do not offer the chance of tracking passerine‐sized birds marked at a stopover site yet. A radiotelemetry network such as the MOTUS Wildlife Tracking System (www.motus.org) could provide useful data without relying on recaptures, but such a system has not been implemented at a large scale in Europe yet. To increase the number of ring recoveries, the only solution is to increase the effort of bird ringing, especially on the breeding grounds. Standardized projects such as the Constant Effort Site (CES) program (Baillie, 1990) offer a good opportunity to check demographics of the local breeding population (Baillie & Schaub, 2009) and increasing the effort will lead to an increase of ringing recoveries to help larger‐scale estimates to be drawn based on migration totals. Integrating data from more sites across the Mediterranean would help to understand trends of populations breeding in areas where monitoring data is scarce or lacking.

Of the species with a significant match in trends between Ponza and the PECBMS index, only five had a positive population trend (Black Redstart, Common Chiffchaff *Phylloscopus collybita*, Blackcap *Sylvia atricapilla*, Subalpine Warbler *Sylvia cantillans*, and Song Thrush *Turdus philomelos*). One species was fluctuating without showing a definite trend (Wryneck *Jynx torquilla*), and 8 species showed decreasing trends (Great Reed Warbler *Acrocephalus arundinaceus*, Tree Pipit *Anthus trivialis*, Barn Swallow, Woodchat Shrike *Lanius senator*, Wood Warbler *Phylloscopus sibilatrix*, Whinchat *Saxicola rubetra*, Turtle Dove *Streptopelia turtur*, Garden Warbler, and Whitethroat). While a discussion of the reasons for these increases or declines is beyond the scope of this study, our results confirm that conservation should be addressed with priority to farmland species and long‐distance migrants (Gregory et al., 2019; Vickery et al., 2014; Voříšek et al., 2010). An interesting example is constituted by the two forest species showing declines, the Garden Warbler and the Wood Warbler. When considering the data from Ponza, our first interpretation of the reduced totals for these two species, which are both very numerous on passage in the island, was the delayed passage during the spring (Maggini, Cardinale et al., 2020) which resulted in a truncated sampling. However, this does not seem to be the case since the trends are similar in the breeding grounds as well. The decrease in these two species might be linked to an increasing mismatch of the timing of arrival and the availability of prey during the breeding season (Both et al., 2006; Møller et al., 2008), or to factors affecting the two species during the non‐breeding season. These two species share a large part of their non‐breeding range (Aymí & Gargallo, 2020; Clement, 2020) and their conservation might deserve increased attention in these areas.

Migratory birds, especially trans‐Saharan migrants, are experiencing worrying population declines (Bairlein, 2016; Vickery et al., 2014), and their conservation needs to address effort in both the breeding and the non‐breeding areas. Monitoring population trends and quickly addressing declines require a huge effort in terms of work force and economically. This study shows that good and immediate estimates of the population processes can be achieved by highly standardized monitoring at migration bottlenecks. This effort needs to be maintained and augmented and could benefit from better assessment of migratory connectivity between stopover and breeding areas. An increase of the ringing effort at the breeding sites, the integration of more long‐term data from migration bottlenecks, as well as future technological developments, will increase the power of estimating population trends that can be used in conservation of bird populations.

## CONFLICT OF INTEREST

The authors declare no competing interest.

## AUTHOR CONTRIBUTION

**Ivan Maggini:** Conceptualization (equal); Writing‐original draft (lead); Writing‐review & editing (equal). **Massimiliano Cardinale:** Conceptualization (equal); Data curation (lead); Formal analysis (lead); Investigation (lead); Methodology (lead); Project administration (equal); Writing‐original draft (supporting); Writing‐review & editing (equal). **Andrea Favaretto:** Formal analysis (supporting); Visualization (lead); Writing‐original draft (supporting); Writing‐review & editing (equal). **Petr Voříšek:** Investigation (supporting); Methodology (supporting); Resources (lead); Writing‐review & editing (equal). **Fernando Spina:** Conceptualization (supporting); Data curation (supporting); Methodology (supporting); Project administration (supporting); Writing‐review & editing (equal). **Francesco Maoret:** Formal analysis (supporting); Writing‐review & editing (equal). **Andrea Ferri:** Investigation (supporting); Writing‐review & editing (equal). **Sara Riello:** Investigation (supporting); Writing‐review & editing (equal). **Leonida Fusani:** Conceptualization (supporting); Funding acquisition (lead); Project administration (supporting); Supervision (lead); Writing‐review & editing (equal).

## Supporting information

Fig S1

Table S1‐S3

Supplementary Material

## Data Availability

The dataset is available at the URL: https://phaidra.vetmeduni.ac.at/view/o:114.
