# Supplementary figures and images for: Comparing population trend estimates of migratory birds from breeding censuses and capture data at a spring migration bottleneck
Source: Ecol Evol. 2020 Dec 19;11(2):967–77. doi: 10.1002/ece3.7110 (PMC7820168; doi:10.1002/ece3.7110)

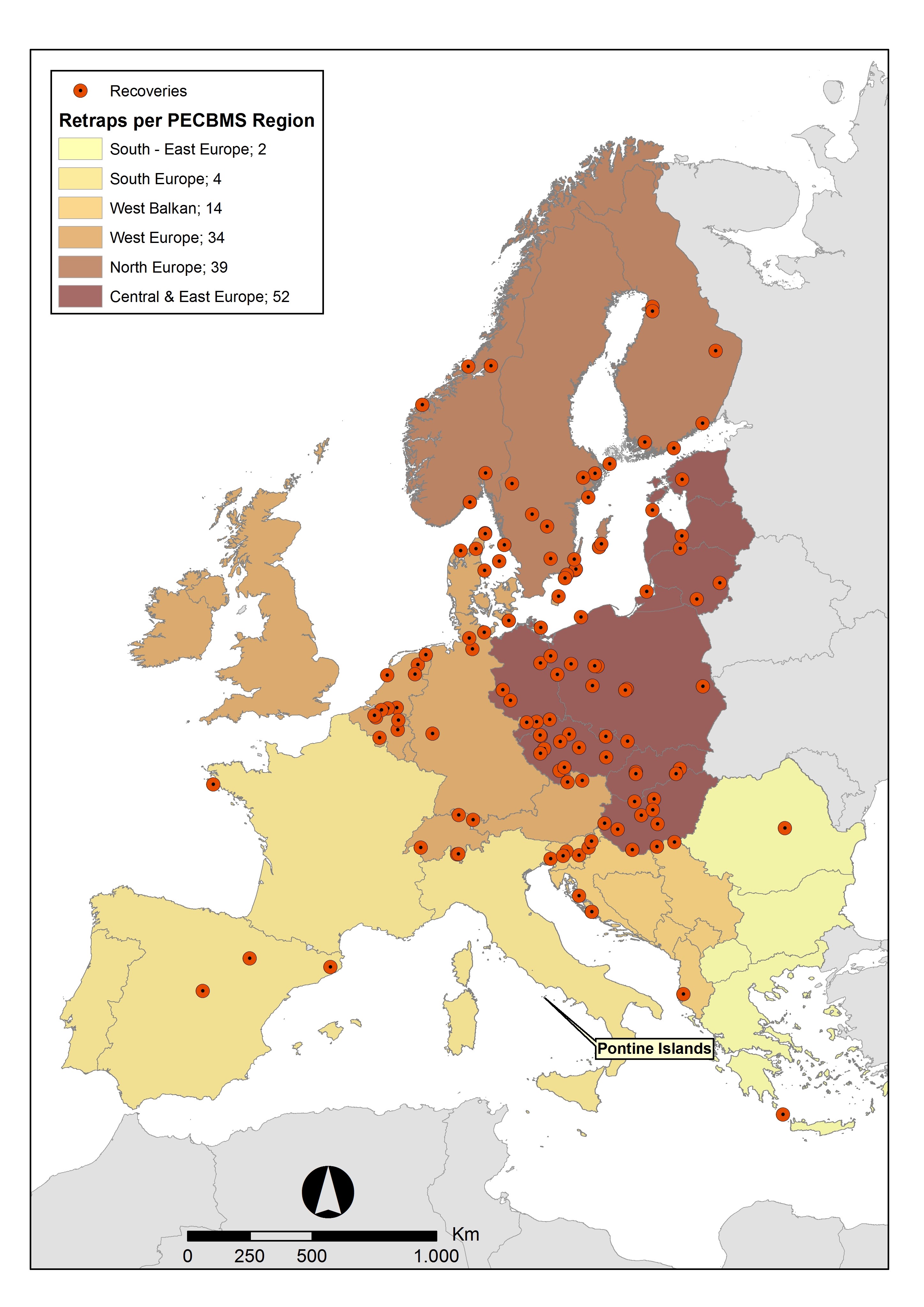

Supplement: Supplementary file 1 — Fig S1 [file ECE3-11-967-s001.jpg]
