# Supplementary material for: Comparing population trend estimates of migratory birds from breeding censuses and capture data at a spring migration bottleneck
Source: Ecol Evol. 2020 Dec 19;11(2):967–77. doi: 10.1002/ece3.7110 (PMC7820168; doi:10.1002/ece3.7110)
Supplement: Supplementary file 2 — Table S1‐S3 [file ECE3-11-967-s002.docx]

Table S1. Start and end date of capture operations on Ponza during the 12 years of the study.

| Year | Start | End |
| --- | --- | --- |
| 2005 | 20 March | 14 May |
| 2006 | 9 April | 13 May |
| 2007 | 26 March | 18 May |
| 2008 | 28 March | 16 May |
| 2009 | 28 March | 15 May |
| 2010 | 27 March | 14 May |
| 2011 | 11 March | 23 May |
| 2012 | 18 March | 24 May |
| 2013 | 11 March | 17 May |
| 2014 | 30 March | 17 May |
| 2015 | 12 March | 22 May |
| 2016 | 13 March | 20 May |

Table S2. List of all recoveries included in the database. We report all recoveries where the capture outside the Pontine Islands was between March 15^th^ and September 20^th^. Recoveries that were not used for the geographical analysis are included with notes on the justification for discarding. *Date, geographical coordinates in decimal degrees and PECBMS region refer to the recovery outside the Pontine islands (Ponza, Ventotene and Zannone).

|  |  |  |  |  |  |  |  |  |  |  |
| --- | --- | --- | --- | --- | --- | --- | --- | --- | --- | --- |
| Species | Ringing station | Ringing country | Retrap station | Retrap country | Latitude* | Longitude* | Date* | PECBMS Region | Discarded | Rationale |
| *Acrocephalus arundinaceus* | Ventotene | Italy | Keszthely-Fenekpuszta | Hungary | 46.76 | 17.25 | 07/06/2000 | Central & East Europe | No |  |
| *Acrocephalus arundinaceus* | Ventotene | Italy | Zb.Wonieść | Poland | 52.00 | 16.70 | 07/18/2007 | Central & East Europe | No |  |
| *Acrocephalus arundinaceus* | Dinnyés | Hungary | Ventotene | Italy | 47.16 | 18.57 | 08/26/2014 | Central & East Europe | No |  |
| *Acrocephalus arundinaceus* | Boguniewo | Poland | Ventotene | Italy | 52.70 | 16.97 | 07/03/1998 | Central & East Europe | No |  |
| *Acrocephalus arundinaceus* | Zannone | Italy | Vrhnika | Slovenia | 45.97 | 14.30 | 08/22/2012 | West Balkan | Yes | Likely migrating |
| *Acrocephalus arundinaceus* | Fenékpuszta | Hungary | Ponza | Italy | 46.71 | 17.25 | 09/13/2006 | Central & East Europe | Yes | Likely migrating |
| *Acrocephalus arundinaceus* | Glibusa | Croatia | Ponza | Italy | 43.07 | 17.65 | 08/11/2006 | West Balkan | Yes | Likely migrating |
| *Acrocephalus schoenobaenus* | Ponza | Italy | Ostrava | Czech Republic | 49.87 | 18.33 | 05/04/2016 | Central & East Europe | No |  |
| *Acrocephalus schoenobaenus* | Ekeren | Belgium | Ventotene | Italy | 51.28 | 4.42 | 08/12/1999 | West Europe | No |  |
| *Acrocephalus schoenobaenus* | Aspvik | Sweden | Ventotene | Italy | 59.48 | 17.68 | 08/20/2007 | North Europe | No |  |
| *Acrocephalus schoenobaenus* | Liminka | Finland | Ponza | Italy | 64.87 | 25.38 | 08/13/2000 | North Europe | No |  |
| *Acrocephalus schoenobaenus* | Vransko jezero | Croatia | Ventotene | Italy | 43.88 | 15.55 | 08/17/2003 | West Balkan | Yes | Likely migrating |
| *Erithacus rubecula* | Ventotene | Italy | Halastó | Hungary | 45.97 | 17.90 | 04/17/2006 | Central & East Europe | No |  |
| *Erithacus rubecula* | Ventotene | Italy | Rönnskär | Sweden | 59.93 | 24.40 | 04/25/2009 | North Europe | No |  |
| *Erithacus rubecula* | Międzyodrze | Poland | Ventotene | Italy | 53.20 | 14.40 | 07/07/2005 | Central & East Europe | No |  |
| *Erithacus rubecula* | Iokva Rovozna | Croatia | Ventotene | Italy | 45.22 | 14.23 | 09/10/2008 | West Balkan | Yes | Likely migrating |
| *Ficedula albicollis* | Rosenburg-Mold | Austria | Ventotene | Italy | 48.63 | 15.62 | 06/30/2002 | West Europe | No |  |
| *Ficedula albicollis* | Kamenec Pod Vtacnikom | Slovakia | Ponza | Italy | 48.67 | 18.55 | 06/03/2014 | Central & East Europe | No |  |
| *Ficedula albicollis* | Rudvier | Sweden | Ponza | Italy | 57.02 | 18.32 | 06/25/2012 | North Europe | No |  |
| *Ficedula albicollis* | Služín | Czech Republic | Zannone | Italy | 49.38 | 17.05 | 06/14/2012 | Central & East Europe | No |  |
| *Ficedula hypoleuca* | Ponza | Italy | Sepanmaki | Finland | 62.87 | 29.38 | 05/13/2011 | North Europe | No |  |
| *Ficedula hypoleuca* | Halloh | Germany | Ventotene | Italy | 53.97 | 9.51 | 06/08/2008 | West Europe | No |  |
| *Ficedula hypoleuca* | Boswachterij | The Netherlands | Ventotene | Italy | 52.63 | 6.26 | 05/24/2011 | West Europe | No |  |
| *Ficedula hypoleuca* | Novi Knezevac | Republic of Serbia | Ponza | Italy | 46.05 | 20.10 | 08/29/2007 | West Balkan | No |  |
| *Ficedula hypoleuca* | Cervenohorské Sedlo | Czech Republic | Ponza | Italy | 50.13 | 17.15 | 08/25/2012 | Central & East Europe | No |  |
| *Hippolais icterina* | Ventotene | Italy | Gedser Odde | Denmark | 54.57 | 11.97 | 08/25/2011 | West Europe | No |  |
| *Hippolais icterina* | Ventotene | Italy | Grønland, Oslo | Norway | 59.92 | 10.77 | 06/17/1993 | North Europe | No |  |
| *Hippolais icterina* | Ventotene | Italy | Augstkalne | Latvia | 56.38 | 23.33 | 06/20/2010 | Central & East Europe | No |  |
| *Hippolais icterina* | Ventotene | Italy | Lichtenberg | Germany | 50.87 | 13.32 | 06/04/2008 | Central & East Europe | No |  |
| *Hippolais icterina* | Ponza | Italy | Sfantu Gheorghe | Romania | 45.85 | 25.78 | 05/13/2014 | SouthEast Europe | No |  |
| *Hippolais icterina* | Zagajew | Poland | Ventotene | Italy | 51.70 | 18.58 | 07/03/2010 | Central & East Europe | No |  |
| *Hippolais icterina* | Ventotene | Italy | P Nac del Archipielago de Cabrera | Spain | 39.58 | 2.65 | 05/13/2009 | South Europe | Yes | Likely migrating |
| *Hirundo rustica* | Ventotene | Italy | Vejvanov | Czech Republic | 49.87 | 13.65 | 06/11/2008 | Central & East Europe | No |  |
| *Hirundo rustica* | Ventotene | Italy | Brunyola | Spain | 41.90 | 2.67 | 08/15/2002 | South Europe | No |  |
| *Hirundo rustica* | Ventotene | Italy | Vransko Jezero | Croatia | 43.88 | 15.55 | 08/30/2012 | West Balkan | No |  |
| *Hirundo rustica* | Ponza | Italy | Locarno | Switzerland | 46.15 | 8.80 | 04/02/2013 | West Europe | No |  |
| *Hirundo rustica* | Ponza | Italy | Heikant 1 | The Netherlands | 51.39 | 5.29 | 07/10/2015 | West Europe | No |  |
| *Hirundo rustica* | Zannone | Italy | Kraghede | Nordjyllands | 57.20 | 10.00 | 06/07/2010 | West Europe | No |  |
| *Hirundo rustica* | Zannone | Italy | Groningen | The Netherlands | 53.35 | 6.87 | 07/28/2013 | West Europe | No |  |
| *Hirundo rustica* | Kolansko Blato - Isola Pag | Croatia | Ventotene | Italy | 44.50 | 14.97 | 08/29/2004 | West Balkan | No |  |
| *Hirundo rustica* | Tauste | Spain | Ventotene | Italy | 41.92 | -1.25 | 06/10/2001 | South Europe | No |  |
| *Hirundo rustica* | Les Grangettes | France | Ventotene | Italy | 46.38 | 6.90 | 08/14/2003 | West Europe | No |  |
| *Hirundo rustica* | Azuqueca de Henares | Spain | Ventotene | Italy | 40.55 | -3.25 | 08/10/2006 | South Europe | No |  |
| *Hirundo rustica* | Pristavica | Slovenia | Ventotene | Italy | 46.20 | 15.65 | 07/21/2002 | West Balkan | No |  |
| *Hirundo rustica* | Izsak (Kolon – TÒ) | Hungary | Ponza | Italy | 46.78 | 19.35 | 08/18/2002 | Central & East Europe | No |  |
| *Hirundo rustica* | Halasto | Hungary | Ponza | Italy | 45.97 | 17.90 | 09/10/2007 | Central & East Europe | No |  |
| *Hirundo rustica* | Bilje | Slovenia | Ponza | Italy | 40.92 | 12.96 | 08/17/2007 | South Europe | No |  |
| *Hirundo rustica* | Aegelsee B. Fauenfeld | Switzerland | Ponza | Italy | 47.57 | 8.87 | 08/24/2007 | West Europe | No |  |
| *Hirundo rustica* | Bokrijk | Belgium | Ponza | Italy | 50.95 | 5.42 | 08/04/2013 | West Europe | No |  |
| *Hirundo rustica* | Verd | Slovenia | Ponza | Italy | 45.97 | 14.30 | 05/03/2014 | West Balkan | No |  |
| *Hirundo rustica* | Vransko Jezero | Croatia | Zannone | Italy | 43.88 | 15.55 | 07/30/2006 | West Balkan | No |  |
| *Hirundo rustica* | Ventotene | Italy | Barcaggio | France | 43.00 | 9.40 | 04/22/2002 | South Europe | Yes | Likely migrating |
| *Hirundo rustica* | Ventotene | Italy | Malia | Greece | 35.29 | 25.49 | 04/15/2012 | SouthEast Europe | Yes | Likely migrating |
| *Hirundo rustica* | Vransko jezero | Croatia | Ventotene | Italy | 43.88 | 15.55 | 09/06/2006 | West Balkan | Yes | Likely migrating |
| *Merops apiaster* | Ventotene | Italy | Vjosa River | Albania | 40.61 | 19.56 | 05/23/2002 | West Balkan | No |  |
| *Merops apiaster* | Edderitz | Germany | Ventotene | Italy | 51.68 | 11.87 | 07/07/2008 | Central & East Europe | No |  |
| *Oenanthe oenanthe* | Ponza | Italy | Klece | Slovenia | 46.12 | 14.50 | 04/22/2014 | West Balkan | No |  |
| *Oriolus oriolus* | Ventotene | Italy | Antikythera | Greece | 35.87 | 23.30 | 05/07/2011 | SouthEast Europe | No |  |
| *Phoenicurus ochruros* | Kos | Greece | Ventotene | Italy | 48.73 | 18.57 | 07/21/2008 | Central & East Europe | No |  |
| *Phoenicurus phoenicurus* | Ventotene | Italy | Joachimsthal | Germany | 52.97 | 13.75 | 05/25/2007 | Central & East Europe | No |  |
| *Phoenicurus phoenicurus* | Ventotene | Italy | Plön | Germany | 54.17 | 10.43 | 06/05/2001 | West Europe | No |  |
| *Phoenicurus phoenicurus* | Ponza | Italy | Tartu | Estonia | 58.77 | 24.47 | 06/01/2013 | Central & East Europe | No |  |
| *Phoenicurus phoenicurus* | Castricum | The Netherlands | Ventotene | Italy | 52.55 | 4.62 | 07/19/2010 | West Europe | No |  |
| *Phoenicurus phoenicurus* | Greifswalder Oie | Germany | Ventotene | Italy | 54.25 | 13.92 | 05/13/2008 | Central & East Europe | No |  |
| *Phylloscopus collybita* | Ventotene | Italy | Nidingen | Sweden | 57.30 | 11.90 | 04/25/2008 | North Europe | No |  |
| *Phylloscopus collybita* | Ventotene | Italy | Hluboka n/Vlt. | Czech Republic | 49.05 | 14.42 | 04/14/2009 | Central & East Europe | No |  |
| *Phylloscopus collybita* | Ventotene | Italy | Bredmar/Byn | Sweden | 58.75 | 17.87 | 05/05/2012 | North Europe | No |  |
| *Phylloscopus collybita* | Ponza | Italy | Nidingen | Sweden | 57.30 | 11.90 | 05/05/2015 | North Europe | No |  |
| *Phylloscopus collybita* | Zannone | Italy | Flakket – Arhus | Denmark | 56.72 | 11.52 | 04/24/2012 | West Europe | No |  |
| *Phylloscopus collybita* | Gedser Odde | Denmark | Ventotene | Italy | 54.57 | 11.97 | 05/26/2005 | West Europe | No |  |
| *Phylloscopus collybita* | Ellekrattet | Denmark | Ventotene | Italy | 57.73 | 10.63 | 04/22/2009 | West Europe | No |  |
| *Phylloscopus collybita* | Koterov | Czech Republic | Ventotene | Italy | 49.72 | 13.43 | 04/08/2006 | Central & East Europe | No |  |
| *Phylloscopus collybita* | Orin | Norway | Ventotene | Italy | 63.78 | 11.43 | 09/16/2008 | North Europe | No |  |
| *Phylloscopus collybita* | Praha 9 | Czech Republic | Ponza | Italy | 50.10 | 14.57 | 07/01/2010 | Central & East Europe | No |  |
| *Phylloscopus collybita* | Nes | Norway | Ponza | Italy | 63.78 | 9.58 | 09/17/2010 | North Europe | No |  |
| *Phylloscopus sibilatrix* | Ventotene | Italy | Harvaluoto | Finland | 60.37 | 22.47 | 06/09/2007 | North Europe | No |  |
| *Phylloscopus sibilatrix* | Ventotene | Italy | Simar Nature Reserve | Malta | 35.95 | 14.38 | 04/29/2009 | South Europe | Yes | Likely migrating |
| *Phylloscopus sibilatrix* | Simar Nature Reserve | Malta | Ponza | Italy | 35.95 | 14.38 | 04/29/2009 | South Europe | Yes | Likely migrating |
| *Phylloscopus trochilus* | Ventotene | Italy | Ottenby | Sweden | 56.24 | 16.45 | 05/20/2004 | North Europe | No |  |
| *Phylloscopus trochilus* | Ventotene | Italy | Hoburgen | Sweden | 56.92 | 18.13 | 05/27/2006 | North Europe | No |  |
| *Phylloscopus trochilus* | Ventotene | Italy | Hoburgen | Sweden | 56.92 | 18.13 | 05/10/2000 | North Europe | No |  |
| *Phylloscopus trochilus* | Ventotene | Italy | Hoburgen | Sweden | 56.92 | 18.13 | 05/31/2007 | North Europe | No |  |
| *Phylloscopus trochilus* | Ventotene | Italy | Sylviastien | Denmark | 57.75 | 10.63 | 05/20/2011 | West Europe | No |  |
| *Phylloscopus trochilus* | Ventotene | Italy | Skultorp | Sweden | 58.35 | 13.88 | 06/02/2010 | North Europe | No |  |
| *Phylloscopus trochilus* | Ventotene | Italy | Vira Bruk | Sweden | 59.57 | 18.55 | 05/31/2014 | North Europe | No |  |
| *Phylloscopus trochilus* | Ventotene | Italy | Lild Strand | Denmark | 57.13 | 8.97 | 08/02/2012 | West Europe | No |  |
| *Phylloscopus trochilus* | Ventotene | Italy | Flakket | Denmark | 56.72 | 11.52 | 04/23/2011 | West Europe | No |  |
| *Phylloscopus trochilus* | Ponza | Italy | Bialka | Poland | 51.38 | 23.00 | 08/01/2011 | Central & East Europe | No |  |
| *Phylloscopus trochilus* | Ponza | Italy | Utklippan | Sweden | 55.95 | 15.70 | 05/02/2014 | North Europe | No |  |
| *Phylloscopus trochilus* | Ponza | Italy | Jakobsbyn | Sweden | 59.50 | 12.63 | 07/19/2012 | North Europe | No |  |
| *Phylloscopus trochilus* | Ottenby | Sweden | Ventotene | Italy | 56.24 | 16.45 | 05/10/2008 | North Europe | No |  |
| *Phylloscopus trochilus* | Ottenby | Sweden | Ventotene | Italy | 56.24 | 16.45 | 04/25/2008 | North Europe | No |  |
| *Phylloscopus trochilus* | Jomfruland | Norway | Ventotene | Italy | 58.88 | 9.61 | 08/15/2004 | North Europe | No |  |
| *Phylloscopus trochilus* | Gedser Odde | Denmark | Ventotene | Italy | 54.57 | 11.97 | 05/15/2008 | West Europe | No |  |
| *Phylloscopus trochilus* | Lebbeke | Belgium | Ventotene | Italy | 51.00 | 4.13 | 07/29/2008 | West Europe | No |  |
| *Phylloscopus trochilus* | Hoburgen | Sweden | Ventotene | Italy | 56.92 | 18.13 | 05/06/1999 | North Europe | No |  |
| *Phylloscopus trochilus* | Zele-Heikant | Belgium | Ventotene | Italy | 51.07 | 4.03 | 08/29/2002 | West Europe | No |  |
| *Phylloscopus trochilus* | Torhamn | Sweden | Ventotene | Italy | 56.08 | 15.85 | 05/16/2007 | North Europe | No |  |
| *Phylloscopus trochilus* | Eriksöre/Karlevi | Sweden | Ventotene | Italy | 56.60 | 16.43 | 07/24/2007 | North Europe | No |  |
| *Phylloscopus trochilus* | Utklippan | Sweden | Ventotene | Italy | 55.95 | 15.70 | 08/16/1996 | North Europe | No |  |
| *Phylloscopus trochilus* | Grimstadvanet | Norway | Ventotene | Italy | 62.37 | 6.00 | 07/27/1999 | North Europe | No |  |
| *Phylloscopus trochilus* | Glinno | Poland | Ventotene | Italy | 51.73 | 18.67 | 07/18/2005 | Central & East Europe | No |  |
| *Phylloscopus trochilus* | Rościnno | Poland | Ventotene | Italy | 52.68 | 17.13 | 06/20/2008 | Central & East Europe | No |  |
| *Phylloscopus trochilus* | Limbach-Oberfrohna | Germany | Ponza | Italy | 50.86 | 12.75 | 08/31/2003 | Central & East Europe | No |  |
| *Phylloscopus trochilus* | Saare | Estonia | Ponza | Italy | 57.92 | 22.05 | 07/27/2003 | Central & East Europe | No |  |
| *Phylloscopus trochilus* | Hoburgen | Sweden | Ponza | Italy | 56.92 | 18.13 | 08/29/2003 | North Europe | No |  |
| *Phylloscopus trochilus* | Greifswalder Oie | Germany | Ponza | Italy | 54.25 | 13.92 | 08/07/2010 | Central & East Europe | No |  |
| *Phylloscopus trochilus* | Wicie | Poland | Ponza | Italy | 54.50 | 16.46 | 04/18/2012 | Central & East Europe | No |  |
| *Phylloscopus trochilus* | Ponza | Italy | Illa de L'aire | Spain | 39.85 | 4.25 | 04/05/2014 | South Europe | Yes | Likely migrating |
| *Phylloscopus trochilus* | R Nat de las Islas Columbretes | Spain | Ventotene | Italy | 39.88 | 0.68 | 04/15/1998 | South Europe | Yes | Likely migrating |
| *Phylloscopus trochilus* | Rio Ebro | Spain | Ventotene | Italy | 41.58 | -0.73 | 08/30/2005 | South Europe | Yes | Likely migrating |
| *Phylloscopus trochilus* | Casares | Spain | Ventotene | Italy | 36.43 | -5.27 | 08/21/2005 | South Europe | Yes | Likely migrating |
| *Phylloscopus trochilus* | Palacios de la Valduerna | Spain | Ventotene | Italy | 42.30 | -5.93 | 09/06/2003 | South Europe | Yes | Likely migrating |
| *Phylloscopus trochilus* | Le Massereau | France | Ponza | Italy | 47.23 | -1.92 | 09/15/2003 | South Europe | Yes | Likely migrating |
| *Saxicola rubetra* | Ventotene | Italy | Greifswalder Oie | Germany | 54.25 | 13.92 | 03/19/2002 | Central & East Europe | No |  |
| *Streptopelia turtur* | Ventotene | Italy | Didymoteicho | Greece | 41.35 | 26.50 | 08/20/2002 | SouthEast Europe | Yes | Likely migrating |
| *Streptopelia turtur* | Ventotene | Italy | Delimara | Malta | 35.82 | 14.57 | 04/27/2001 | South Europe | Yes | Likely migrating |
| *Streptopelia turtur* | Ventotene | Italy | Rhenia | Greece | 37.37 | 25.37 | 09/03/2001 | SouthEast Europe | Yes | Likely migrating |
| *Streptopelia turtur* | Ventotene | Italy | Zebbug | Malta | 35.87 | 14.43 | 04/30/2011 | South Europe | Yes | Likely migrating |
| *Streptopelia turtur* | Ventotene | Italy | Gurgulyat | Bulgaria | 42.78 | 22.98 | 08/18/2007 | SouthEast Europe | Yes | Likely migrating |
| *Streptopelia turtur* | Ventotene | Italy | Kilkis | Greece | 40.75 | 22.98 | 08/30/2014 | SouthEast Europe | Yes | Likely migrating |
| *Streptopelia turtur* | Zannone | Italy | Shkodër | Albania | 42.07 | 19.52 | 04/23/2011 | West Balkan | Yes | Likely migrating |
| *Sylvia atricapilla* | Ventotene | Italy | Sächsische Schweiz | Germany | 50.92 | 14.07 | 04/23/2009 | Central & East Europe | No |  |
| *Sylvia atricapilla* | Ventotene | Italy | Wanzleben | Germany | 52.07 | 11.45 | 05/21/2010 | Central & East Europe | No |  |
| *Sylvia atricapilla* | Ponza | Italy | Horusice | Czech Republic | 49.15 | 14.70 | 04/24/2011 | Central & East Europe | No |  |
| *Sylvia atricapilla* | Ponza | Italy | Wedel | Germany | 53.58 | 9.70 | 04/23/2014 | West Europe | No |  |
| *Sylvia atricapilla* | Ralangen | Sweden | Ventotene | Italy | 57.87 | 14.85 | 08/30/2008 | North Europe | No |  |
| *Sylvia atricapilla* | Awirs | Belgium | Ventotene | Italy | 50.60 | 5.40 | 09/02/2007 | West Europe | No |  |
| *Sylvia atricapilla* | Berzée | Belgium | Ventotene | Italy | 50.28 | 4.40 | 09/09/1989 | West Europe | No |  |
| *Sylvia atricapilla* | D. Jesenice | Slovenia | Ventotene | Italy | 45.95 | 15.13 | 09/14/2006 | West Balkan | No |  |
| *Sylvia atricapilla* | Nimtofte | Denmark | Ventotene | Italy | 56.40 | 10.53 | 04/20/2011 | West Europe | No |  |
| *Sylvia atricapilla* | Lustenau | Austria | Ventotene | Italy | 47.40 | 9.65 | 09/09/2007 | West Europe | No |  |
| *Sylvia atricapilla* | Niederbreitbach | Germany | Ventotene | Italy | 50.51 | 7.38 | 08/30/2004 | West Europe | No |  |
| *Sylvia borin* | Ventotene | Italy | Szalonna (AH) | Hungary | 48.46 | 20.71 | 08/22/2008 | Central & East Europe | No |  |
| *Sylvia borin* | Ponza | Italy | Ottenby | Sweden | 56.20 | 16.40 | 08/12/2002 | North Europe | No |  |
| *Sylvia borin* | Ponza | Italy | Laajakoskenjarvi | Finland | 60.53 | 26.85 | 06/13/2003 | North Europe | No |  |
| *Sylvia borin* | Ponza | Italy | Saltoniskes | Lithuania | 54.87 | 25.29 | 07/03/2013 | Central & East Europe | No |  |
| *Sylvia borin* | Ponza | Italy | Naszaly | Hungary | 47.68 | 18.30 | 08/27/2013 | Central & East Europe | No |  |
| *Sylvia borin* | Ponza | Italy | Christianso | Denmark | 55.32 | 15.19 | 05/18/2013 | West Europe | No |  |
| *Sylvia borin* | Zannone | Italy | Ocsa (AH) | Hungary | 47.68 | 19.35 | 08/11/2014 | Central & East Europe | No |  |
| *Sylvia borin* | Jomfruland | Norway | Ventotene | Italy | 58.88 | 9.61 | 09/09/2002 | North Europe | No |  |
| *Sylvia borin* | Hoburgen | Sweden | Ventotene | Italy | 56.92 | 18.13 | 09/02/2003 | North Europe | No |  |
| *Sylvia borin* | Hoburgen | Sweden | Ventotene | Italy | 56.92 | 18.13 | 08/15/2006 | North Europe | No |  |
| *Sylvia borin* | Riečiai | Lithuania | Ventotene | Italy | 54.48 | 23.68 | 08/15/2004 | Central & East Europe | No |  |
| *Sylvia borin* | Hajdoše | Slovenia | Ventotene | Italy | 46.42 | 15.83 | 08/23/2008 | West Balkan | No |  |
| *Sylvia borin* | Drienovec | Slovakia | Ventotene | Italy | 48.62 | 20.95 | 09/09/2007 | Central & East Europe | No |  |
| *Sylvia borin* | Oulu | Finland | Ponza | Italy | 65.02 | 25.48 | 08/12/2001 | North Europe | No |  |
| *Sylvia borin* | Ocsa (AH) | Hungary | Ponza | Italy | 47.30 | 19.21 | 08/20/2007 | Central & East Europe | No |  |
| *Sylvia borin* | Szalonna (AH) | Hungary | Ponza | Italy | 48.46 | 20.71 | 08/16/2011 | Central & East Europe | No |  |
| *Sylvia borin* | Ventotene | Italy | Vrhnika | Slovenia | 45.97 | 14.30 | 08/20/2006 | West Balkan | Yes | Likely migrating |
| *Sylvia borin* | Ventotene | Italy | Vransko Jezero | Croatia | 43.88 | 15.55 | 08/28/2009 | West Balkan | Yes | Likely migrating |
| *Sylvia borin* | Ponza | Italy | Vrhnika | Slovenia | 45.97 | 14.30 | 08/21/2008 | West Balkan | Yes | Likely migrating |
| *Sylvia borin* | Ponza | Italy | Wied L-Ahmar | Malta | 36.00 | 14.30 | 04/30/2016 | South Europe | Yes | Likely migrating |
| *Sylvia borin* | Vrhnika | Slovenia | Ventotene | Italy | 45.97 | 14.30 | 08/18/2004 | West Balkan | Yes | Likely migrating |
| *Sylvia borin* | Vrhnika | Slovenia | Ventotene | Italy | 45.97 | 14.30 | 09/12/2008 | West Balkan | Yes | Likely migrating |
| *Sylvia borin* | Vrhnika | Slovenia | Ventotene | Italy | 45.97 | 14.30 | 08/19/2009 | West Balkan | Yes | Likely migrating |
| *Sylvia borin* | Vnanje Gorice | Slovenia | Ventotene | Italy | 46.00 | 14.42 | 08/18/2002 | West Balkan | Yes | Likely migrating |
| *Sylvia borin* | Bevke | Slovenia | Ventotene | Italy | 45.98 | 14.37 | 08/12/2007 | West Balkan | Yes | Likely migrating |
| *Sylvia borin* | Vransko jezero | Croatia | Ventotene | Italy | 43.88 | 15.55 | 08/25/2004 | West Balkan | Yes | Likely migrating |
| *Sylvia borin* | Vransko jezero | Croatia | Ventotene | Italy | 43.88 | 15.55 | 08/19/2007 | West Balkan | Yes | Likely migrating |
| *Sylvia borin* | Vransko jezero | Croatia | Ventotene | Italy | 43.88 | 15.55 | 08/20/2006 | West Balkan | Yes | Likely migrating |
| *Sylvia borin* | Retje | Slovenia | Ventotene | Italy | 45.77 | 14.38 | 08/22/2006 | West Balkan | Yes | Likely migrating |
| *Sylvia borin* | Slovenska Vas | Slovenia | Ventotene | Italy | 45.97 | 15.07 | 08/30/2007 | West Balkan | Yes | Likely migrating |
| *Sylvia borin* | Vrhnika | Slovenia | Ponza | Italy | 45.97 | 14.30 | 08/17/2002 | West Balkan | Yes | Likely migrating |
| *Sylvia borin* | Vransko Jezero | Croatia | Ponza | Italy | 43.88 | 15.55 | 09/01/2008 | West Balkan | Yes | Likely migrating |
| *Sylvia borin* | Buskett | Malta | Ponza | Italy | 35.85 | 14.43 | 05/11/2006 | South Europe | Yes | Likely migrating |
| *Sylvia borin* | Verd | Slovenia | Ponza | Italy | 45.97 | 14.30 | 08/23/2008 | West Balkan | Yes | Likely migrating |
| *Sylvia borin* | Dragomer | Slovenia | Ponza | Italy | 46.02 | 14.35 | 08/20/2010 | West Balkan | Yes | Likely migrating |
| *Sylvia borin* | Cap D'antibes Einlenroc | France | Ponza | Italy | 43.55 | 7.13 | 05/11/2012 | South Europe | Yes | Likely migrating |
| *Sylvia borin* | Vrhnika | Slovenia | Ponza | Italy | 45.97 | 14.30 | 08/19/2012 | West Balkan | Yes | Likely migrating |
| *Sylvia borin* | Zalosce | Slovenia | Ponza | Italy | 45.88 | 13.73 | 09/11/2012 | West Balkan | Yes | Likely migrating |
| *Sylvia borin* | Wied L-Ahmar | Malta | Ponza | Italy | 36.00 | 14.30 | 04/30/2013 | South Europe | Yes | Likely migrating |
| *Sylvia borin* | Vransko Jezero | Croatia | Ponza | Italy | 43.88 | 15.55 | 09/12/2011 | West Balkan | Yes | Likely migrating |
| *Sylvia borin* | Jezero, Njivice | Croatia | Ponza | Italy | 45.17 | 14.57 | 08/18/2012 | West Balkan | Yes | Likely migrating |
| *Sylvia borin* | Vransko Jezero | Croatia | Ponza | Italy | 43.88 | 15.55 | 08/20/2016 | West Balkan | Yes | Likely migrating |
| *Sylvia communis* | Ventotene | Italy | Rybachiy Fringilla | Russia | 55.09 | 20.73 | 05/31/2009 | Central & East Europe | No |  |
| *Sylvia communis* | Ventotene | Italy | Jomfruland | Norway | 58.88 | 9.61 | 05/16/1990 | North Europe | No |  |
| *Sylvia communis* | Ventotene | Italy | Zabice | Slovenia | 52.52 | 14.72 | 05/17/2009 | Central & East Europe | No |  |
| *Sylvia communis* | Ventotene | Italy | Högnalöv | Sweden | 56.70 | 14.88 | 05/22/2008 | North Europe | No |  |
| *Sylvia communis* | Ventotene | Italy | Sovolusky | Czech Republic | 50.33 | 15.12 | 04/25/2001 | Central & East Europe | No |  |
| *Sylvia communis* | Ventotene | Italy | Golczewice | Poland | 52.85 | 15.57 | 06/11/2012 | Central & East Europe | No |  |
| *Sylvia communis* | Ponza | Italy | Lagskar | Finland | 59.83 | 19.70 | 05/28/2002 | North Europe | No |  |
| *Sylvia communis* | Ponza | Italy | Spacice | Czech Republic | 49.82 | 15.60 | 07/16/2009 | Central & East Europe | No |  |
| *Sylvia communis* | Ponza | Italy | Lycklamavaart | The Netherlands | 52.98 | 6.41 | 07/22/2012 | West Europe | No |  |
| *Sylvia communis* | Zannone | Italy | Bad Grosspertholz | Niederösterreich | 48.62 | 14.81 | 05/23/2009 | West Europe | No |  |
| *Sylvia communis* | Bolle Di Magadino | Switzerland | Ventotene | Italy | 46.16 | 8.86 | 05/02/2006 | West Europe | No |  |
| *Sylvia communis* | Körmend | Hungary | Ventotene | Italy | 47.02 | 16.60 | 08/24/2010 | Central & East Europe | No |  |
| *Sylvia communis* | Kalnciems | Latvia | Ventotene | Italy | 56.80 | 23.60 | 08/05/2002 | Central & East Europe | No |  |
| *Sylvia communis* | Ventotene | Italy | Illa de L'Aire | Spain | 39.85 | 4.25 | 05/04/2008 | South Europe | Yes | Likely migrating |
| *Sylvia communis* | Vransko Jezero | Croatia | Ponza | Italy | 43.88 | 15.55 | 09/02/2009 | West Balkan | Yes | Likely migrating |
|  |  |  |  |  |  |  |  |  |  |  |
| *Upupa epops* | Ventotene | Italy | Stroupeč | Czech Republic | 50.35 | 13.50 | 07/12/2013 | Central & East Europe | No |  |
| *Upupa epops* | Ventotene | Italy | Privlaka | Croatia | 50.37 | 13.47 | 06/15/2012 | Central & East Europe | No |  |
|  |  |  |  |  |  |  |  |  |  |  |

Table S3. Mean centroid coordinates estimated for species with at least three recoveries and for all species together using data from Pontine Islands and assigned to each of the PECBMS region taken between 1989 and 2016 during the breeding period.
