# Supplementary material for: Comparing population trend estimates of migratory birds from breeding censuses and capture data at a spring migration bottleneck
Source: Ecol Evol. 2020 Dec 19;11(2):967–77. doi: 10.1002/ece3.7110 (PMC7820168; doi:10.1002/ece3.7110)
Supplement: Supplementary file 3 — Supplementary Material [file ECE3-11-967-s003.docx]

**Figure S1** Spatial distribution of the recoveries from Pontine Islands in each of the PECBMS regions between 1989 and 2016 during the breeding period.
